# A pilot study of the association between maternal mid-pregnancy cholesterol and oxysterol concentrations and labor duration

**DOI:** 10.1186/s12944-023-01800-8

**Published:** 2023-03-11

**Authors:** Todd C. Rideout, Jaclyn Wallace, Xiaozhong Wen, Vanessa M. Barnabei, Kai Ling Kong, Richard W. Browne

**Affiliations:** 1 Department of Exercise and Nutrition Sciences, School of Public Health and Health Professions, Jacobs School of Medicine and Biomedical Sciences, University at Buffalo, Buffalo, NY 14214 USA; 2 Department of Biotechnical and Clinical Laboratory Sciences, Jacobs School of Medicine and Biomedical Sciences, University at Buffalo, Buffalo, NY 14214 USA; 3 Department of Pediatrics, Jacobs School of Medicine and Biomedical Sciences, University at Buffalo, Buffalo, NY 14214 USA; 4 Department of Obstetrics and Gynecology, Jacobs School of Medicine and Biomedical Sciences, University at Buffalo, Buffalo, NY 14214 USA; 5 Baby Health Behavior Lab, Division of Health Services and Outcomes Research, Children’s Mercy Research Institute, Children’s Mercy Hospital, Kansas City, USA; 6 Department of Pediatrics, University of Missouri, Columbia, USA; 7 Center for Children’s Healthy Lifestyles and Nutrition, University of Kansas Medical Center, Kansas City, KS USA

**Keywords:** Cholesterol, Oxysterols, Labor dystocia, Labor duration, Pregnancy

## Abstract

**Background:**

Previous animal model studies have highlighted a role for cholesterol and its oxidized derivatives (oxysterols) in uterine contractile activity, however, a lipotoxic state associated with hypercholesterolemia may contribute to labor dystocia. Therefore, we investigated if maternal mid-pregnancy cholesterol and oxysterol concentrations were associated with labor duration in a human pregnancy cohort.

**Methods:**

We conducted a secondary analysis of serum samples and birth outcome data from healthy pregnant women (*N* = 25) with mid-pregnancy fasting serum samples collected at 22–28 weeks of gestation. Serum was analyzed for total-C, HDL-C, and LDL-C by direct automated enzymatic assay and oxysterol profile including 7α-hydroxycholesterol (7αOHC), 7β-hydroxycholesterol (7βOHC), 24-hydroxycholesterol (24OHC), 25-hydroxycholesterol (25OHC), 27-hydroxycholesterol (27OHC), and 7-ketocholesterol (7KC) by liquid chromatography-selected ion monitoring-stable isotope dilution-atmospheric pressure chemical ionization-mass spectroscopy. Associations between maternal second trimester lipids and labor duration (minutes) were assessed using multivariable linear regression adjusting for maternal nulliparity and age.

**Results:**

An increase in labor duration was observed for every 1-unit increment in serum 24OHC (0.96 min [0.36,1.56], *p* < 0.01), 25OHC (7.02 min [1.92,12.24], *p* = 0.01), 27OHC (0.54 min [0.06, 1.08], *p* < 0.05), 7KC (8.04 min [2.7,13.5], *p* < 0.01), and total oxysterols (0.42 min [0.18,0.06], *p* < 0.01]. No significant associations between labor duration and serum total-C, LDL-C, or HDL-C were observed.

**Conclusions:**

In this cohort, mid-pregnancy concentrations of maternal oxysterols (24OHC, 25OHC, 27OHC, and 7KC) were positively associated with labor duration. Given the small population and use of self-reported labor duration, subsequent studies are required for confirmation.

## Background

An increase in maternal lipids during pregnancy is recognized as a normal physiological response to pregnancy, with large increases in triglycerides (~ 200–400%), total-cholesterol (~ 25–50%), and low-density lipoprotein (LDL-C, ~ 70%) beginning in early pregnancy and peaking in the third trimester [1, 2]. However, recent work has highlighted concerns that an abnormal dyslipidemic profile during pregnancy may have negative health implications for both the mother and fetus [3, 4]. We previously reported that total particle concentrations of very low-density lipoprotein/chylomicron remnants (VLDL/CM) and high-density lipoproteins (HDL) in mid-pregnancy have divergent associations with birth weight [5]. In this brief report, we extend our focus to examine the association between maternal pregnancy cholesterol and oxysterol concentrations and labor duration.

Labor dystocia, defined as an abnormally slow or protracted labor [6], increases the risk of both maternal and neonatal complications [7, 8] and is a primary indication for cesarean delivery [6]. Predictive maternal biomarkers of a labor dystocia phenotype may be useful in identifying at-risk pregnancies and initiating early monitoring and prevention strategies. A potential link between lipotoxicity and labor dystocia has been suggested by previous human and animal model studies demonstrating a critical role for cholesterol in successful parturition but impaired uterine function and contractile activity in hypercholesterolemia states [9–12].

Several regulatory proteins controlling myometrial contraction, including the oxytocin receptor (OXTR) and human ether-a-go-go-related gene (hERG) potassium channel proteins [13] are localized in cholesterol-rich microdomains (lipids rafts and caveolae) and are therefore sensitive to local and circulating cholesterol concentrations [10, 14]. The OXTR expresses a cholesterol binding site which is important for receptor stabilization and posttranslational processing [15–17]. Further, oxysterols, a class of highly reactive oxygenated derivates of cholesterol that induce inflammation have been shown to function as nuclear receptor ligands to regulate intracellular cholesterol concentration in the mouse uterus [18]. However, despite the known function of cholesterol and its oxidative derivatives in regulating uterine contractile activity, we are not aware of any previous human cohorts that have examined if associations exist between maternal sterol status during pregnancy and labor duration. Therefore, in this pilot study, we sought to examine if maternal mid-pregnancy cholesterol and oxysterol concentrations were associated with labor duration.

## Methods

### Participants

We undertook a secondary analysis of serum samples and birth outcome data collected as part of a previous pregnancy pilot cohort study. All procedures were approved by the Institutional Review Board at the University at Buffalo (STUDY00001381). Details on the study design and cohort have previously been published [5]. Briefly, the cohort consisted of 25 adult pregnant women aged 18–35 years with singleton gestations recruited within the greater Buffalo, New York area between 2018 and 2019 through flyer distribution, social media posts, and local urban obstetrics clinics (Table 1). Women were excluded if they had a history of major chronic diseases (e.g., heart disease, diabetes) or were currently taking medications that affected appetite or insulin sensitivity. None of the mothers included in this cohort had premature infants or cesarean deliveries.

**Table 1:** Characteristics of mothers in the pilot cohort

Variable (***N*** = 25)	Mean (SD)	Median (IQ)	n (%)	Range
**Age (yrs)**	30.7 (3.96)			21.6–37.8
**Race**				
White			23 (92)	
African American			1 (4.0)	
Asian			1 (4)	
**Education**				
Level (yrs)	16.1 (2.1)			
College graduate			23 (92)	
**Pre-Pregnancy BMI (kg/m**^**2**^**)**^**a**^				
Normal			10 (40)	
Overweight			7 (28)	
Obese			8 (32)	
Nulliparous			16 (64)	
**Length of active labor (hrs)**		7.0 (9.0)		0.67–30
**Labor**				
Labor induction			8 (32)	
Use of pain medication^b^			19 (76)	
**Gestational age**^**c**^		39 (1.0)		32–41
**Birth weight (g)**	3543 (333)			
**Mid-Pregnancy Serum Lipids (mg/dL)**				
Total-C	250.6 (49.3)			131–374
LDL-C	141.7 (41.1)			40–250
HDL-C	78.9 (20.8)			33–117
Triglycerides	172.8 (61.4)			87–398
**Mid-Pregnancy Serum Oxysterols (ng/mL)**^**d**^				
24-hydroxycholesterol (24OHC)	84.9 (29.3)			(42.8–146.9)
25-hydroxycholesterol (25OHC)	18.4 (3.5)			(10.7–27.0)
27-hydroxycholesterol (27OHC)	144.5 (39.3)			(66.9–211.7)
7⍺-hydroxycholesterol (7⍺OHC)	47.4 (27.0)			(13.4–122.2)
7β-hydroxycholesterol (7βOHC)	7.6 (5.0)			(0.06–21.3)
7-ketocholesterol (7 KC)	5.0 (2.9)			(1.2–12.3)
Total oxysterols^a^	307.2 (72.0)			(145.9–473.9)

^a^Normal BMI 18.5–24.9; overweight BMI 25–29.9; obese BMI 30–34.9

^b^Including epidural analgesia (*n* = 16) and narcotic pain medications (*n* = 3)

^c^Gestational weeks at time of birth

^d^Represents the sum of 24OHC, 25OHC, 27OHC, 7⍺OHC, 7βOHC, and 7KC

### Data collection

At enrollment, prospective women completed an interviewer-administered questionnaire to obtain information regarding general socio-demographics as well as reproductive and medical history. Maternal height and pre-pregnancy weight were collected by self-report at the initial visit. Body mass index (BMI) was calculated as pre-pregnancy weight in kilograms divided by the square of height in meters. At mid-pregnancy, between gestation weeks 22–28, body weight and height were directly measured by research staff using a digital scale and digital stadiometer, respectively. At this same visit, a fasting blood sample was collected by a blood draw technician. The total duration of active labor (> 4 cm dilation) was obtained with a self-reported birth outcome questionnaire assessed at a home visit within 7 days of birth.

### Serum lipid analyses

Following a blood clotting period of 30 minutes at room temperature, serum samples were separated by centrifugation at 3000 x g for 20 min and stored at − 80 °C. Total-cholesterol (TC), low-density lipoprotein cholesterol (LDL-C), and high-density lipoprotein cholesterol (HDL-C) were measured by direct automated enzymatic assay using diagnostic reagent kits, calibrators, and quality controls.

Serum oxysterols including 7α-hydroxycholesterol (7αOHC), 7β-hydroxycholesterol (7βOHC), 24-hydroxycholesterol (24OHC), 25-hydroxycholesterol (25OHC), 27-hydroxycholesterol (27OHC), and 7-ketocholesterol (7KC) were determined by liquid chromatography-selected ion monitoring-stable isotope dilution-atmospheric pressure chemical ionization-mass spectroscopy (LC-SIM-SID-APCI-MS) as previously described [19, 20] with slight modification. Briefly, serum oxysterols were released from steryl esters by incubation with microbial cholesterol esterase (100ul containing 2 U for 20 minutes at 37 °C) and following solid phase extraction, were injected into an Ascentis 10 cm X 3 mm, 3-μm C-18 LC column (SupelCo Inc.) on a Shimadzu 10ADVP LC system interfaced with a Shimadzu 2010A MS by APCI interface. Deuterated (d7) 22HC, (d7) 7αHC and (d7) 7KC was used as stable, isotope-labeled, internal standards. We, and others [21], have found that oxysterol analytes are stable at -80 °C storage. The assay was previously validated in accordance with the FDA guidance for bioanalytical methods [22].

### Statistical analyses

*The r*elationship of individuals oxysterols with serum cholesterol and maternal BMI were assessed with Pearson correlation coefficients. Associations between maternal second trimester lipids and labor duration were assessed using multivariable linear regression. As labor duration data was not normally distributed, the variable was log transformed for all analyses. We considered nulliparity (yes/no), birth weight, gestation age, maternal age and pre-pregnancy BMI (continuous variables) as potential confounding factors. Confounders with a (*p*-value < 0.05) were considered significant and were further adjusted in the models. Two models were used in the analysis: (i) Model 1 was the crude model with second trimester lipid exposure only; and (ii) Model 2 adjusted for maternal nulliparity and maternal age (the only two significant confounders). All analyses were conducted using SAS version 9.4 (SAS Institute, Inc., Cary, NC). Statistical significance was set at a two-sided alpha level of 0.05. A post hoc power analysis, based on linear bivariate regression between maternal total oxysterols and labor duration, with significance set at α = 0.05, yielded a power (1-β) of 0.766 (G*Power 3.1.5) [23].

## Results

Mothers included in this study were mainly White (92%), highly educated (92% college graduate), and overweight (28%) or obese (32%) before pregnancy (Table 1). The majority of women 16 (64%) in the cohort were nulliparous. Mean active labor duration was 8.9 ± 6.9 hours (13.9 ± 8.6 hours for nulliparous women; 6.2 ± 4.2 hours multiparous women).

Serum oxysterols were not correlated (*p* > 0.05) with maternal BMI at pre-pregnancy or mid-pregnancy (Table 2). Serum 24OHC (*r* = 0.37), 27OHC (*r* = 0.49), 7KC (*r* = 0.33), and total oxysterols (*r* = 0.52) were positively correlated with total-C, however, no correlation (*p* > 0.05) was observed between cholesterol and 25OHC, 7⍺OHC, and 7βOHC. Amongst the oxysterols, only 27OHC and total oxysterols had significant (*p* < 0.05) positive associations with LDL-C (*r* = 0.43 and 0.41) and HDL-C (*r* = 0.51 and 0.42).

**Table 2:** Correlations between maternal mid-pregnancy oxysterol concentrations, weight status and serum lipids

		Mid-Pregnancy Serum Oxysterols (ng/mL)						
Maternal characteristic (***n*** = 25)		24OHC	25OHC	27OHC	7⍺OHC	7βOHC	7 KC	Total Oxysterols^a^
**Weight status**								
Pre-pregnancy BMI (kg/m^2^)	**r**	−0.19	−0.13	− 0.21	− 0.02	− 0.04	0.00	− 0.20
	**p**	0.31	0.31	0.27	0.94	0.81	1.00	0.27
Mid-pregnancy BMI (kg/m2)	**r**	−0.15	−0.11	− 0.15	0.03	− 0.08	0.05	− 0.14
	**p**	0.42	0.42	0.41	0.88	0.66	0.80	0.44
**Serum Lipids**								
Total-C (mg/dL)	**r**	0.37	0.19	0.49	0.23	−0.15	0.33	0.52
	**p**	0.04	0.30	0.004	0.21	0.41	0.07	0.002
LDL-C (mg/dL)	**r**	0.28	0.20	0.43	0.14	−0.22	0.21	0.41
	**p**	0.12	0.26	0.01	0.45	0.23	0.25	0.02
HDL-C (mg/dL)	**r**	0.22	0.10	0.51	0.10	−0.10	0.32	0.42
	**p**	0.23	0.60	0.00	0.57	0.60	0.07	0.02

r, Pearson Correlation Coefficient; *p*, *p*-value; ^a^Represents the sum of 24OHC, 25OHC, 27OHC, 7⍺OHC, 7βOHC, and 7KC

No significant associations between labor duration and serum total-C (*p* = 0.08), LDL-C (*p* = 0.07), and HDL-C (*p* = 0.93) were observed (Table 3). Among the oxysterols, with the exception of 7⍺OHC and 7βOHC, an increase in labor duration was observed for every 1-unit increment in serum serum 24OHC (0.96 min [0.36,1.56], *p* < 0.01), 25OHC (7.02 min [1.92,12.24], *p* = 0.01), 27OHC (0.54 min [0.06, 1.08], *p* < 0.05), 7KC (8.04 min [2.7,13.5], *p* < 0.01), and total oxysterols (0.42 min [0.18,0.06], *p* < 0.01] (Table 3).

**Table 3:** Associations between maternal oxysterol concentrations and labor duration

	Labor Duration (minutes)			
Serum lipids	Model 1 β (95% CI) ***N*** = 25	***p***-value	Model 2 β (95% CI) ***N*** = 25	***p***-value
**Lipid panel (mg/dL)**				
Total-cholesterol	0.36 (−0.18,1.02)	0.22	0.48 (−0.06,0.96)	0.08
LDL-cholesterol	0.48 (−0.36,1.32)	0.25	0.60 (−0.06,1.32)	0.07
HDL-cholesterol	0.06 (−1.2,1.26)	0.97	−0.06(−1.02,0.9.6)	0.93
**Oxysterols (ng/mL)**				
24-hydroxycholesterol	1.14 (0.42,1.86)	< 0.01	0.96 (0.36,1.56)	< 0.01
25-hydroxycholesterol	6.9 (0.24,13.62)	0.04	7.02 (1.92,12.24)	0.01
27-hydroxycholesterol	0.72 (0.06,1.32)	0.03	0.54 (0.06,1.08)	0.04
7⍺-hydroxycholesterol	0.24 (−0.48,1.08)	0.50	0.42 (− 0.18,1.02)	0.20
7β-hydroxycholesterol	1.56 (−3.36,6.48)	0.53	1.02 (−3.06,5.1)	0.62
7-ketocholesterol	7.38 (0.42,14.34)	0.03	8.04 (2.7,13.5)	< 0.01
Total oxysterols	0.48 (0.24,0.72)	< 0.01	0.42 (0.18,0.06)	< 0.01

*Values are beta coefficients (95% CI) derived from linear regression models, representing the change in labor duration (minutes) with one unit increment in each lipid exposure; Model 1: crude model; Model 2: adjusted for maternal nulliparity and age

## Discussion

In this cohort, maternal mid-pregnancy serum oxysterol concentrations (24OHC, 25OHC, 27OHC, and 7KC) were positively associated with labor duration. However, the magnitude of these associations varied depending on the oxysterol species, possibly reflecting their specific routes of synthesis and functional roles. Interestingly, serum TC and LDL-C were not associated with labor duration. This contrast is perhaps surprising given the significant correlations we observed between serum total oxysterols and cholesterol and the known role of oxysterols in regulating intracellular cholesterol efflux and accumulation [18]. Alternatively, due to the specific roles of oxysterols as inflammatory mediators and nuclear receptor ligands, oxysterols may represent a more sensitive marker of uterine contraction than cholesterol.

Oxysterols are highly reactive oxygenated derivatives of cholesterol that differ in their oxidation sites (within the A or B rings or the side chain of cholesterol) and their routes of synthesis [24]. They can be generated through specific cytochrome P450 mitochondrial or microsomal enzymes that are coordinately regulated through substrate and co-factor availability or through random non-enzymatic autooxidation routes involving reactive oxygen species [25]. Oxysterols of enzymatic origin, including 7αOHC, 24OHC, 25OHC, and 27OHC, are most often identified as intermediates of bile acid synthesis, however, they also act as intracellular ligands for several nuclear receptors and thus play a functional role in regulating a broad range of metabolic pathways, including cholesterol metabolism. Alternatively, oxysterols of non-enzymatic origin, including 7βOHC and 7KC, are typically recognized for their pro-oxidant and pro-inflammatory biological activities through their induction of inflammatory cytokine expression [24].

Although oxysterols have been implicated in the pathogenesis of several disease states including atherosclerosis [26] and dementia [27], few studies have characterized oxysterol concentrations during pregnancy or examined their relationship with pregnancy outcomes. In a longitudinal pregnancy cohort of 33 women, Winkler et al. (2017) [28] reported an increase in maternal 27OHC throughout pregnancy but no association with several pregnancy complications including intrauterine growth restriction (IUGR), preeclampsia (PE), and hemolysis, elevated liver enzymes, low platelet count (HELLP) syndrome. However, Mistry et al. observed an increase in placental 27OHC concentration and enhanced maternal and fetal cholesterol-mediated serum efflux capacity in pregnancies affected by preeclampsia compared with normotensive controls [29]. A potential role of oxysterols in placental dysfunction is further supported through in vitro studies reporting the pro-inflammatory actions of 25OHC and 7KC through a toll-like receptor 4 (TLR4)-dependent mechanism [30] and cytotoxic effects of 25OHC in placental trophoblasts [31].

We are not aware of previous human studies that have examined the association between maternal cholesterol or their oxygenated derivatives and labor duration. The observed differences in the relative magnitude of the effect for individual oxysterol species may be of interest in their potential utility as predictive biomarkers of labor dystocia. At this point, the underlying reasons for these differential responses are not known. However, while the physiological effects of oxysterols as a class of compounds has been historically emphasized [32], more recent work has recognized the metabolic implications of individual oxysterol species which may be influenced by several factors including absolute concentrations and tissue distribution [33], differential ligand binding affinities [34], and a diverse range of metabolic activities [24].

Although our findings cannot provide causative evidence to support a direct role of maternal sterol status on labor duration, results from animal model studies suggest several mechanistic links between lipotoxicity (specifically, cholesterol and oxysterols) and labor dystocia. First, as the precursor substrate of steroid hormones, cholesterol is essential for the endocrine balance between estrogen and progesterone that activates the myometrium and regulates the onset of labor [35]. Second, under normal physiological concentrations, cholesterol is involved in the stabilization and posttranslational processing of the oxytocin receptor [15, 16], however, an abnormal increase in cholesterol has been shown to blunt oxytocin-induced contractile activity in mouse [12] and human [11] uterine tissue. Third, previous work by Mouzat et al. (2006) in mice suggests that oxysterols may have a role in preserving uterine contractile function under excessive cholesterol conditions by signaling increased transcription of cholesterol efflux genes through ligand activation of the liver X receptor β (LXRβ) [18].

The results of this pilot study should be interpreted with caution given several study limitations, First, due to resource constraints, this study included 25 pregnant women of which only 64% were nulliparous and for whom labor dystocia is more commonly diagnosed. However, we hope the results provide insight into estimated effect size for the design of future randomized controlled trials to identify lipid biomarkers of labor dystocia. Second, our sample was limited with respect to race/ethnicity and education and may be biased due to potentially inaccurate assessment of labor duration with a self-reported birth outcome questionnaire. Finally, oxysterol analysis was limited to mid-pregnancy only, and it is unknown how concentrations at this timepoint correlate with those at early and late gestation phases.

## Conclusions

In this cohort, mid-pregnancy concentrations oxysterols (24OHC, 25OHC, 27OHC, and 7KC) were positively associated with labor duration. However, considering the small population and use of self-reported labor duration, subsequent studies are required to support the potential use of serum oxysterols as biomarkers of labor duration.

## Data Availability

The datasets used and/or analyzed during the current study are available from the corresponding author on reasonable request.

## Abbreviations

OXTR: Oxytocin receptor
hERG: Human ether-a-go-go-related gene
BMI: Body mass index
TC: Total-cholesterol
LDL-C: Low-density lipoprotein cholesterol
HDL-C: High-density lipoprotein cholesterol
7αOHC: 7α-hydroxycholesterol
7βOHC: 7β-hydroxycholesterol
24OHC: 24-hydroxycholesterol
25OHC: 25-hydroxycholesterol.
27OHC: 27-hydroxycholesterol
7KC: 7-ketocholesterol
LC-SIM-SID-APCI-MS: Liquid chromatography-selected ion monitoring-stable isotope dilution-atmospheric pressure chemical ionization-mass spectroscopy
IUGR: Intrauterine growth restriction
PE: Preeclampsia
HELLP: Hemolysis, elevated liver enzymes, low platelet count syndrome
TLR4: Toll-like receptor 4
LXRβ: Liver X receptor β
